# Experiences of parenting for autistic children in Australia and Italy: a qualitative cross-cultural comparison

**DOI:** 10.3389/fpsyg.2024.1409234

**Published:** 2024-08-05

**Authors:** Alessandra Frigerio, Tess Knight, Riccardo Rella, Elena Sinigaglia, Elisabetta Camussi, Lorenzo Montali

**Affiliations:** ^1^Department of Psychology, Sigmund Freud University, Milan, Italy; ^2^Cairnmillar Institute, Hawthorn East, VIC, Australia; ^3^Department of Psychology, University of Milano-Bicocca, Milan, Italy

**Keywords:** parenting, autism, cross-cultural research, qualitative research, Australia, Italy

## Abstract

**Introduction:**

Parents vary in their gradual adaptation to the demands of caring for an autistic child. Their experiences can be positive, negative, or a combination of both, depending on the severity of the cases and various personal and social factors. Cross-cultural comparisons can aid in understanding how contextual factors, such as the availability of social and health services, and cultural factors, such as family relationships and the distribution of care burden, may influence the caregiving experience. However, there is a limited amount of cross-cultural research on this topic, which is almost exclusively quantitative.

**Aims and methods:**

From an ecocultural perspective, our interview-based study aimed to explore the experiences of Australian (15) and Italian (11) parents of autistic children living in their respective countries.

**Results:**

The analysis identified three main themes. *Life through autism, autism through life: coexisting with an unexpected life partner*, *Parenting autistic children: reorganization of parents’ subjectivity and familiar dynamics*, and *The unseen side of disability: autism communities between inclusion and segregation*.

**Discussion:**

Our comparative approach reveals both commonalities and differences between the two groups of participants. The similarities support features previously identified in the literature: the emotional and physical burden on parents and the positive impact on parental identity. The differences relate to the diagnostic process, interactions with health professionals, the impact of autism on family dynamics and parental perspectives, and the role assigned to siblings. Despite facing similar challenges, these differences reveal how Italian and Australian parents articulate different understandings linked to social and cultural differences.

## Introduction

Autism spectrum disorder (ASD) is defined as a range of developmental disorders with an onset typically occurring in early childhood. These disorders are characterized by varying but persistent difficulties in social communication and social interaction and restricted, repetitive patterns of behavior, interests, or activities (American Psychiatric Association, 2022). The DSM-5 introduced a new classification system for autism spectrum disorder, which encompasses three previously distinct disorders: autism, Asperger’s disorder, and childhood disintegrative disorder. The reclassification of these disorders as part of a single entity has led to a re-evaluation of the concept of ASD as a spectrum, prompting a debate about the implications of this for the boundaries of the diagnosis. Psychodynamic approaches have historically emphasized the presumed role of parent–child dysfunctional interactive patterns in the condition’s etiology (Avdi et al., 2000). However, the neurological, biogenetic, and cognitive paradigms have supplanted the association of ASD with poor parenting, establishing themselves as the foundation for contemporary ASD understanding.

Caring for an autistic child can be exhausting and challenging (Sarriá and Pozo, 2015). In various forms and degrees of intensity, this challenge characterizes the entire life of the child and the family, from the onset of the first symptoms through adulthood (Gray, 2002). Families might need to modify their daily lives to better meet their children’s needs (Glazzard and Overall, 2012). Also, it generally hinders or impedes the family’s leisure and recreational activities and its involvement in the community (Dillenburger et al., 2010). For these reasons, studies converge on the relevance of parental burden, physical exhaustion, and stress that emerge when comparing parents with ASD children and parents whose children are undiagnosed or those with other clinical syndromes (Mulligan et al., 2012). A meta-synthesis of 25 qualitative studies notes that the parenting experience is characterized by emotional, informational, and relational needs, highlighting the wide range of issues involved (Legg and Tickle, 2019). Research also shows that parents are concerned about their unmet needs and about planning for the future (Dillenburger et al., 2010). The combination of these characteristics has led some authors to equate this parental experience with chronic trauma (Stewart et al., 2016, 2017).

In a study conducted by Casey et al. (2012), 256 parents (245 mothers) were surveyed to investigate whether elevated stress in parents of children with ASD could be conceptualized within a traumatic stress framework. Although these parents did not meet the full criteria for a DSM-IV-TR diagnosis of post-traumatic stress disorder (PTSD), one in five parents reported experiencing severe adverse reactions that were indicative of trauma-related symptomatology. The reactions mentioned above were identified by the authors as subclinical levels of PTSD and designated “post-traumatic stress symptoms” (PTSS). The clinical features of PTSS included intrusion or re-experiencing of the diagnosis (e.g., involuntary thoughts about the event), avoidance (e.g., avoiding reminders of the diagnosis), and hyperarousal (e.g., a constant feeling of being easily startled). In a recent Australian study, Stewart et al. (2020) investigated the validity of conceptualizing elevated stress in parents of children who exhibit challenging behavior within the framework of post-traumatic stress disorder. The study included 395 parents (379 mothers) of children with autism spectrum disorder (*n* = 226), parents of children with a rare disease (*n* = 139), and parents of typically developing (TD) children (*n* = 30). A significantly higher prevalence of PTSD symptomatology was observed among parents of children with ASD and parents of children with a rare disease compared to parents of typically developing children. Furthermore, the presence of challenging child behaviors was identified as a positive predictor of PTSD symptomatology in both groups. A subgroup of parents with autistic children who participated in this study was then recruited for a new study in which they were interviewed using the gold standard diagnostic interview for post-traumatic stress disorder. The findings provided diagnostic evidence of PTSD in parents of children with autism spectrum disorder relating to Criterion A for PTSD traumatic stressors specific to parenting a child with ASD (Schnabel et al., 2020).

However, other studies showed that some families have explicitly asserted the normality of their lives with their autistic children, focusing on their children’s abilities and strengths (Potter, 2016; Halstead et al., 2018; Cost et al., 2021). Research also shows that many families cope well with the additional demands of parenting an autistic child and achieve a good balance between the needs of the child and those of other family members (Higgins et al., 2023). Indeed, families differ in their gradual adjustment to the condition, and their experiences can be described as positive, negative, or a combination of both, depending on the severity of the cases (Nurullah, 2013). These differences depend on several factors: financial resources, the availability of social support and appropriate services, the characteristics of the autistic child, the coping strategies parents can develop, and their perceptions of their problems (Sarriá and Pozo, 2015).

Qualitative research can help to explore these different family trajectories and how they are constructed in relation to the socio-cultural context in which parents live (Nurullah, 2013). Doing so can also show how the diversity of experiences and discourses produced by parents contribute to the construction of autism as a product of interrelated social and biological processes (Belek, 2019).

The specificity of our research is the possibility of comparing family experiences within a cross-cultural logic, highlighting similarities and differences between two different contexts, Australia and Italy. This comparison does not attempt to identify contextual features that have a deterministic effect on the construction of these experiences, nor would this be possible in a qualitative study. Instead, we seek to show how the meanings people attach to their experiences take shape within their relationships with the contexts in which they live.

In the following pages, we first discuss the literature identifying the key critical issues in the parenting experience at different stages of a child’s life. We then present the studies that have looked at the strategies parents use to cope with caring and the narratives they use to explain their pathways and choices, and we discuss similarities and differences between the two countries that are relevant to the scope of this study.

### A long-life story of difficulties and challenges

The first difficulty parents face is in the pre-diagnosis period, in the early years of the child’s life, when parents notice differences in the child’s speech, communication, and behavior (Boshoff et al., 2021). Parents observe various warning signs or report that others have commented on their child’s behavior, and their struggle to make sense of the situation is typically characterized by confusion and feelings of emotional overwhelm (Legg and Tickle, 2019). Parents’ uncertainty can also be exacerbated by family members and/or health and education professionals dismissing their suspicions and stating nothing is wrong (Singh, 2016).

In turn, the process of obtaining a diagnosis is very stressful for parents for several reasons. It is a long path because it usually involves meetings with professionals who offer various alternatives to autism diagnosis and because of the waiting times for multidisciplinary assessments (Boshoff et al., 2021). Parents often feel that they are not adequately listened to by uncooperative clinicians and report that a diagnosis is difficult to obtain (Singh, 2016). The general dissatisfaction with the diagnostic process described in the literature is also due to the content of the diagnostic and ASD information shared and how clinicians communicate this information (Mulligan et al., 2012). As a result, parents’ reports are characterized by feelings of shock, relief, anxiety, grief, anger, isolation, guilt, and uncertainty at receiving a diagnosis (Legg and Tickle, 2019).

Parents may also experience conflict about the usefulness of a diagnosis. They know the label allows them to access a range of care services and build a pathway within the social and health system to benefit and support their child (Legg and Tickle, 2019). Nevertheless, they may also fear that the diagnosis of autism could lead to prejudice and discrimination against their child or themselves (Glazzard and Overall, 2012). Furthermore, in some mothers’ narratives, this transition is depicted “by employing the trope of an abrupt crossing over from the ‘normal’ to the ‘abnormal’“(Lilley, 2011, p. 209) to signify a loss of the original hopes and expectations they had for their children (O’Brien, 2007). Indeed, the diagnosis is a constitutive act, not simply a descriptive label, which inscribes the child in a category that is both enabling and constraining and which assigns a new role to the parent and redefines the parent–child relationship (Atwood and Gallo, 2010).

Parents’ difficulties are exacerbated by the fact that the period following the diagnosis is often characterized by a perceived lack of support for them and their children (Altiere and Von Kluge, 2009). For example, parents feel that they are not given clear indications for treatment as there are no specific drugs, and they may be offered different intervention strategies (Singh, 2016). Moreover, parents may be overwhelmed by the frequently required role of co-therapist (Pakenham et al., 2005). They also complain that they are left alone to identify available services and resources, a remarkably complex task as there is no reference point to help parents untangle different structures, each with its bureaucracy (Blum, 2007). The limited economic resources of families and the lack of appropriate services and social support generally contribute to a poor prognosis (Dillenburger et al., 2010). Even in schools, parents feel they have to face teachers who are often unaware of the impact of having an autism diagnosis on the families’ lives (Dillenburger et al., 2004) and often lack sufficient expertise in the therapies the children are involved in (Keenan et al., 2007). Parents’ concerns are reinforced by the media’s portrayal of life with autism as a tragedy made of limitations, troubles, and problems that are difficult to overcome (Lilley, 2011).

In the relational sphere, parents experience a narrowing of their friendship network when they face situations requiring more emotional and social support. This is partly because the child’s needs take up most of their time, forcing them to reduce their social space (Yirmiya and Shaked, 2005). It is also due to the social stigma that surrounds ASD and leads to the social isolation of these parents (Silverman, 2008). The social rejection mainly affects the mothers who, according to a still prevalent traditional view, are expected to bear much of the burden of care (Lilley, 2013).

### Trajectories of familiar adjustment and resistance

Longitudinal research, on the other hand, shows that the story of families can be described as the progressive development of an adaptation to disability that goes through several stages, the course and outcome of which may be positive or at least less harmful than in the beginning (Gray, 2002). Indeed, families’ resilience may enable them to cope with the challenges outlined above by responding appropriately to the additional demands of parenting an autistic child and reducing their stress (Gerstein et al., 2009). This is done primarily by developing a range of coping strategies, some of which are also used by parents of children with other disabilities (Gray, 2006; Frigerio and Montali, 2016). These strategies include relying on family or support groups to compensate for the lack of support from other networks, avoiding certain social situations that may be a source of distress, or turning to faith to make sense of their challenges (Silverman, 2008). Developing these strategies also makes it possible to strengthen the threatened sense of control and thus contribute to the overall well-being of families (Barnett et al., 2003). Among these coping resources, advocacy plays a unique role. It can be defined as a set of behaviors aimed at obtaining care for one’s child, promoting the child’s well-being and that of the family as a whole, as well as the child’s rights, and informing others about the child’s developmental condition (Boshoff et al., 2016). These strategies make it possible to transform negative emotions into positive outcomes, as they help to make the demands of these families visible positively and proactively and promote social change (Dababnah and Parish, 2013).

Research also shows that relationships with extended family, especially grandparents, can improve over time and that parents can expand their network of social relationships by interacting with other families with the same disability (Gray, 2002). These two factors help to reduce social isolation.

Capturing these dimensions means trying to understand how people learn over time to give meaning and coherence to their world and their experience of care (Gray, 2001). For example, Gentles and colleagues showed a four-step narrative used by parents to explain their understanding of their child’s autism: “forming an image of difference,” “starting to question the sign,” “knowing something is wrong,” and “being convinced it is autism” (2020; p. 476). These narratives may draw on the hegemonic neuropsychological discourse, which becomes a common-sense framework for understanding oneself and others, endowed with legitimacy and credibility (Ortega and Choudhury, 2011). In other cases, parents may instead resist the professional autism discourse and generate counter-narratives that express parental competence, which is a source of family resilience (Lilley, 2011). For example, narratives to conceptualize notions of enabling care can take different forms: ‘the linear narrative’, based on an individual adjustment model of disability; ‘the narrative of challenge’, expressing resistance to normative and oppressive interpretations of disability; or ‘the philosophy of present and becoming’, a narrative that embraces uncertainty with a sense of optimism and hope (Fisher and Goodley, 2007).

These narratives also allow parents to produce specific positioning of their children and themselves. For example, in some maternal narratives, the representation of their children is focused on their capacity to love, empathy, and compassion, emphasizing their imagination, playfulness, and reflexive self-awareness (Lilley, 2011). Similarly, parents may highlight their child’s sense of humor, social and communication skills, or ability to care for others and do things for themselves (Singh, 2016). In this manner, mothers can position their children by enhancing their specificity and individuality (Lilley, 2011). This emphasis on the distinctive attributes of one’s child does not negate the value of the insights gained from diagnostic categories, the utility of which is acknowledged. In fact, it illustrates how parents can synthesize diverse knowledge and employ it flexibly, contingent upon the circumstances. In turn, mothers position themselves as capable caregivers based on the expertise they have accumulated through their daily interactions with their children (Lilley, 2011).

### A transcultural perspective on caregiving for an autistic child

Although ASD is a widespread condition globally, its prevalence varies over time and across cultures due to cultural influences affecting the identification of symptoms, diagnosis, and treatment (Zeidan et al., 2022). For this reason, a cross-cultural comparison can help understand how contextual factors relating to the availability of social and health services and cultural factors relating to family relationships and the distribution of the care burden may influence their caregiving experience.

Cross-cultural studies on this topic are limited. Recent research has revealed differences in parenting stress levels between participants from individualistic cultures, such as Italy, and collectivist cultures, such as Japan (Giannotti et al., 2021). A study was conducted to investigate the quality of parenting life in seven countries, namely Australia, Hungary, Malaysia, Romania, Singapore, Spain, and the United Kingdom (Eapen et al., 2023). The study found that rankings between countries on each domain differed markedly, indicating cultural differences in how QoL is built. A study conducted in the United States examined the relationship between resilience and parenting stress for autistic children in African American, Hispanic, and White families (Kim et al., 2020). The study found that culture significantly influenced this relationship, even when families were living in the same geographical location and facing similar challenges in caring for their autistic children.

Research proposing cross-cultural comparisons of the impact of caregiving an autistic child is almost exclusively quantitative, which limits our knowledge of parents’ experiences as it cannot represent the nuances of individual voices, emotions, and perspectives (Anderson and Clarke, 2019). Our study aims to address this gap in the scientific literature, investigating the potential differences between Australia and Italy in how autism is represented and lived by parents.

Comparatively, both countries have public health systems financed by national and local taxes, which cover hospital care, medical services, and pharmaceuticals (Tikkanen et al., 2020). Australia spends 10.3% of its GDP on health care, while Italy spends 8.8% (Tikkanen et al., 2020). Although, to the best of our knowledge, no study directly compared the quantity and quality of services provided to people with autism in the two countries, some data suggest differences. In Australia, the National Disability Insurance Scheme (NDIS) provides funding for disability-related services and supports, including individualized interventions for children with autism (Rasheed, 2023). Studies have shown that mental health services in Australia have better accessibility and higher quality, particularly in dedicated early support for autism, compared to other nations (United Kingdom National Health Service, 2021). In Italy, autism is included in the standard healthcare provision provided to all citizens by the National Health System (Borgi et al., 2022). The Ministry of Health and the regions have agreed on strategic priorities for ASD healthcare to promote harmonized protocols for ASD diagnosis and evidence-based intervention approaches aligned with international standards. However, regions have autonomy in deciding how to structure their health systems within this general framework. Some studies suggest that national programs are necessary to improve the structural capacity of the healthcare sector and address inequalities within the country (Borgi et al., 2019).

The diagnosis of ASD in both countries usually requires a comprehensive assessment by a team of qualified professionals, including pediatricians, psychologists, speech therapists, and occupational therapists. The process involves gathering information from parents, caregivers, and teachers and observing the child’s behavior and development (Rasheed, 2023). In both countries most children with special needs, including those with autism, are integrated into the regular school system (Roberts and Webster, 2022; Scattoni et al., 2023).

Similarities and differences can be observed between the two countries regarding characteristics that may affect the burden of family care. One common element is that both countries have an individualistic culture that prioritizes promoting their child’s uniqueness (Giannotti et al., 2021). This is because personal characteristics are expected to impact individual development more than group expectations and needs.

Referring to the concept of familism may be useful to provide context for the Italian situation. Familism is the practice of relying on one’s family for social protection, with minimal intervention from the state (Ruspini, 2015). Some authors argue that familism is a product of a culture rooted in traditional values (Dalla Zuanna, 2004), while others suggest that it results from the limited availability of public services and benefits (Gribaudi, 1997). Familism in Italy has resulted in a distinct gender regime with informal rules. Females are expected to take on the role of caretakers in a traditional family setting, while a single-earner family is encouraged (Camussi et al., 2023a). The importance placed on the family is also evident in the relationships between its members. Research indicates that the strong bond between parents and children is a characteristic of the Italian model (Reher, 2004). The Italian cultural system and welfare model continue to rely on traditional gender roles, the expectation of family support, and women’s unpaid care work (Tobío, 2017). Furthermore, financial ties between generations are often extended (Tomassini et al., 2003).

In Australia, as in other Anglo countries, there is a normative view that young children require constant and sustained parental attention, particularly among the middle classes (Craig and Mullan, 2011). The primary responsibility for child-rearing still falls on women, indicating that parenting of young children is still perceived as a feminine role due to the persistence of traditional gender views (Hunt et al., 2015). However, cross-cultural studies have revealed generally progressive gender attitudes and legislation that enshrine equal opportunities in the workplace (Craig and Mullan, 2011). In this context, fathers’ contribution to childcare was highest in Australia and lowest in other European countries, including Italy. The distribution of time between paid and unpaid work among Australian parents remains gendered, with fathers typically in full-time paid employment and mothers often in part-time employment or not employed at all (Craig et al., 2012). In the Italian case, however, the imbalance is even more pronounced, given that female employment rates are among the lowest among OECD countries (Severini et al., 2019).

In conclusion, this overview highlights similarities and differences between Italy and Australia regarding health services and social and cultural aspects, which are relevant to this research.

### Aims

This study aimed to explore the experiences of parents of autistic children, in particular the meanings they attribute to the problems they face, their experiences specific to the functioning of the family system, and the perceived social meanings attributed to autism.

Understanding parents’ experiences with autism can provide important notions for developing tailored interventions and support as well as improve the quality of health services for families. Understanding parents’ perspectives may also provide relevant information for teachers, health professionals, and the larger community where the children and their families live.

Further, our study explores participants’ caregiving experiences from an ecocultural perspective (Weisner, 2002). We hope to understand how the conceptualizations of problems Italian and Australian parents perceive might vary across different socio-cultural conditions (Kim, 2012).

## Materials and methods

### Procedure

In order to recruit participants, we contacted some organizations in Italy and Australia that provide support to parents of children with autism. In Italy, the research was introduced to the associations’ referees via email, and subsequently, in a meeting, they were asked to disseminate the call for participation through their mailing list. In Australia, we contacted the moderators of Facebook parental groups to request permission to advertise the research via a post presenting the study on the Facebook pages. Additionally, a flyer was created and displayed on the noticeboards of consenting clinicians with expertise in autism spectrum disorder. However, this method proved unsuccessful in terms of participant recruitment.

In both countries, parents who consented to participate were sent an email containing an outline of the project and a consent form. The participants were requested to identify an appropriate time and location for the interviews, which were conducted in person in Italy and via telephone in Australia. No incentives were provided to the participants for their involvement in the study. The participants were furnished with the interview transcript to read and review prior to analysis. This enabled them to ascertain that the most pertinent aspects of their experience had been captured and potentially to provide additional information.

### Participants

To be eligible to participate in the study, respondents must have been aged over 18 years and have a child diagnosed with Autistic Spectrum Disorder. The sample consisted of 26 parents, 11 from Italy and 15 from Australia. Tables 1, 2 summarize the participants’ demographic characteristics and some information about their children and their diagnosis.

**Table 1:** Characteristics of the Australian sample.

**Participants ID**	**Gender**	**Age**	**Level of education**	**Employment status**	**Marital status**	**Child age**	**Child gender**	**Year of diagnosis**	**Sibling**
AU1	F	36	Master degree	Part-time	Married	9	M	2014	Yes
AU2	F	47	Bachelor degree	Full-time	Married	14	M	2005	No
AU3	F	31	Graduate Diploma	Full-time	Married	3	F	2016	No
AU4	F	36	Graduate Diploma	Part-time	Married	3 and 6	M/F	2016	Yes
AU5	F	46	Graduate Diploma	Self-employed	Married	13	M	2007	Yes
AU6	F	45	Master degree	Part-time	Married	14 - twins	M/F	2006	Yes
AU7	M	47	Bachelor degree	Self-employed	*De facto*	8	M	2011	No
AU8	F	44	Bachelor degree	Part-time	Married	8	M	2014	Yes
AU9	F	45	Master degree	Part-time	Married	12	M	2008	Yes
AU10	M	45	Master degree	Full-time	Married	12	M	2008	Yes
AU11	F	42	Undergraduate	Unemployed	Married	14	F	2007	Yes
AU12	F	49	High school diploma	Full-time	Married	19	F	2002	Yes
AU13	F	42	Master degree	Casually	Married	12	M	2015	Yes
AU14	F	53	Master degree	Part-time	Separated	14	M	2011	Yes
AU15	F	46	Master degree	Part-time	Divorced	16	M	2003	Yes

**Table 2:** Characteristics of the Italian sample.

**Participant ID**	**Gender**	**Age**	**Level of education**	**Employment status**	**Marital status**	**Child age**	**Child gender**	**Year of diagnosis**	**Sibling**
ITA1	F	40	High school diploma	Part-time	Separated	7	F	2010	No
ITA2	F	42	Master degree	Full-time	Married	12	F	2005	Yes
ITA3	F	47	Secondary school diploma	Homemaker	Married	16	M	2006	No
ITA4	F		High school diploma	Homemaker	Married	21	M	1997	No
ITA5	F	49	High school diploma	Full-time	Divorced	10	M	2011	No
ITA6	F	36	Master degree	Homemaker	Married	3	M	2013	Yes
ITA7	F	51	High school diploma	Homemaker	Married	15	M	2005	No
ITA8	M	51	High school diploma	Full-time	Married	16	M	2006	No
ITA9	M	55	High school diploma	Full-time	Married	22	M	1995	No
ITA10	M	48	High school diploma	Full-time	Married	14	F	2005	Yes
ITA11	F	41	High school diploma	Self-employed	Married	17	M	2012	Yes

### Ethics

The Ethics Committee of the relevant Universities in each country approved the project. Data were stored in password-protected files on the Universities’ servers. All identifying information was removed from the interview transcripts before the analysis. In the informed consent signed by participants, we indicated counseling services that parents might contact after the interview should they wish to do so.

### Data collection

Interviews with Italian parents were conducted in person or via Skype if face-to-face meetings were impossible. The data collection took place in 2014 over eight months. In Australia, interviews were conducted via telephone to maximize participation. Recruitment for the study occurred over four months, from December 2016 to March 2017.

The interviews were conducted in a semi-structured manner with a schedule that included several questions that were used as a guide. The topic guide was developed based on the research aims and previous literature. The interview protocol is shown in Appendix A.

The interviews were all conducted by the first author and lasted between approximately 30 and 80 min; they were audio-recorded to be verbatim and transcribed for analysis.

### Data analysis

Data were analyzed using a predominantly inductive and semantic form of reflexive Thematic Analysis (Braun and Clarke, 2006, 2019; Clarke and Braun, 2018), in which relevant themes were identified, focusing on the respondents’ meanings. The specific research questions concerning the psychological and social impacts of autism on parents informed our data-driven analysis. Accordingly, we elected to adopt an experiential orientation to investigate how autism was experienced and lived by participants, with a particular emphasis on their thoughts and points of view (Braun and Clarke, 2021).

An iterative approach to data analysis was taken whereby a preliminary analysis of the interviews with Italian parents was conducted to identify codes and then themes. Once a coding framework was collaboratively defined, it was then used to guide the analysis of the Australian data, which were collected later.

The analysis yielded a thematic map comprising six themes, which the research group subsequently revised. The revision entailed the integration of two overlapping themes pertaining to the subjectivity of parents and motherhood, the incorporation of a theme into another pertaining to the meanings attributed to living with autism, and the exclusion of a theme concerning the positioning of specialists and the school context, as our focus was on parental experience rather than on how they position other social actors. The comparison between the two samples led to the definition of a revised final list of common themes, which the research team considered capturing both similarities and differences in the experiences of Italian and Australian parents.

Reflecting on what underpinned this research, we understand that in approaching this field of research, we were moved by an interest in what characterizes the interface between clinical issues and social factors. Understanding how cultural and environmental factors may constrain personal experiences of clinical and health conditions can support a more comprehensive and ecological approach to complex experiences (Colucci and Montali, 2008; Montali et al., 2023). Reflecting on the assumptions embedded in our construction and reading of data, we became aware of how much our psychological disciplinary tradition has informed how we view data—for example, setting the expectation that autism represents a troubled situation and a challenge for parents. As discussed in the following section, our results show that parental experiences are not reducible to this narrow lens but are much more diversified and less compact than expected.

All the data were analyzed in their original language. The quotes from Italian interviews were first translated from Italian to English and then reviewed to ensure equivalence of meaning. The translation was undertaken by one of the authors, a native Italian speaker with proficiency in English. The individual responsible for translating the quotations is also the person who conducted all the interviews in Italy and in Australia. She also has experience with ASD at both academic and clinical levels. The translated quotations were then subjected to a review by a professional bilingual (Italian/English) translator.

## Results

We identified three main themes, each organized in a number of sub-themes.

The theme, *Life through autism, autism through life: coexisting with an unexpected life partner,* concerns the path towards the diagnosis and the general experience of autism, including the adjustment parents had to make because of it. The theme, *Parenting autistic children: reorganization of parents’ subjectivity and familiar dynamics,* regards the impact of autism on the family’s organization and the parents’ subjectivity. The theme, *The unseen side of disability: autism communities between inclusion and segregation,* is about the impact of autism on social life and its implications within the social realm.

After each quote, the participant’s origin is indicated in brackets by their country code (AUS for Australia or ITA for Italy) followed by their corresponding ID number in the interview database.

### Life through autism, autism through life: coexisting with an unexpected life partner

#### What is going on with my child? Autism before the diagnosis

For the majority of the Italian participants, the signs parents noticed before obtaining a diagnosis were described as a set of senseless, indefinable, and inexplicable traits. What parents saw when they looked at their children was something they found extremely difficult to name, represent, and communicate to others. In this regard, Italian participants most often described their children’s manifestations as ‘strangeness,’ ‘abnormality,’ ‘diversity,’ ‘peculiarity,’ and ‘oddity.’ However, many parents were unable to identify specific symptomatic characteristics; they most often saw something wrong with respect to age-normative standards:

*Understanding her peculiarity has not always been easy because she had some oddities, some things … she wasn't growing up in a harmonic way as she should have grown up (…) in kindergarten they told me that she used to do things that she shouldn't do at her age (ITA3)*

The word “harmonic” in the above quote shows how respect for normative standards is directly linked to the perception of proper functioning and ideas of equilibrium and aesthetic goodness.

In contrast, Australian parents tended to circumscribe and limit the areas they perceived as problematic, signifying strangeness into lists of specific behaviors referring to codified diagnostic criteria:

*Probably when he was in kindergarten, we started to notice that he was very particular about some things and he wasn't great socially. So, he would've been about four at the time maybe and he was very much into putting things into specific order. He was a bit picky with his food, he didn't like food being mixed up, chopped up and (…) with his food he'd like it all separate, he didn't like it touching and things like that (…) he was very particular about that (AU14)*

Thus, language use, repeated behavioral patterns, poor social interaction and oversensitivity to environmental stimuli represent the medical-oriented lens through which parents get to see and understand their children, as professionals do.

In both groups, the atypical character of the children’s behavior was characterized as an excess of precocity, a sort of over-human condition that allows children to do things that go beyond usual human limits and cause astonishment to dazed parents:

*He had an impressive characteristic as he crawled and ran! He crawled faster than the children who were already walking, he crawled fast (…) I said: "Oh God, aren't you hurt?" (…) the first oddity we noticed was that (ITA8)*

*She already showed some strange abilities, for example she learned to read by herself, I don't know how she did it (…) one evening I pass by and I say fuck, but she is...she is reading completed things, but nobody had told her A, B, C you have to read (..) she learned by herself (AU11)*

Parents highlight that being out of average, these perceived over-human abilities go beyond every usual expectation, causing a sense of the unexpected that is associated with anxiety and concern.

#### The relief of knowing, the struggle of starting an unexpected journey

In both groups, most parents felt relief after getting the diagnosis. The diagnostic label gives the possibility to name an undefinable strangeness, relieving parents of feelings of inadequacy and guilt.

*I was very relieved (…) because I spent two years of hell where nobody said … they all said it was me and he was normal like all the other children but it's not true, he wasn't normal like all the children (ITA6)*

*I was relieved that I wasn't paranoid, and I took it as a fairly positive thing because I felt that would have been more of an instruction book and a great way of finding out how we could help [child's name] (AU12)*

The diagnosis is taken, on the one hand, as a clear and unquestionable sign of the difference with respect to “all the children” and, on the other hand, as a sort of standardized “instruction book” including procedural intervention models. The feeling of relief, however, goes with several struggles that were mainly related to the relationship with health professionals and services.

Italian parents tended to complain about the single professionals, who were seen as lacking knowledge and expertise in the field of autism:

*I saw one of our pediatricians after I got the diagnosis, and she told me, "mm, you know much, much more than me" ok, but you are the doctor, honey! If you don't' activate your brain, we're not doing well (ITA7)*

Parental expectations that doctors knew more than them were frequently disappointed.

*The child psychiatrist said nothing was wrong with him, so we felt reassured. It's not that we don't trust doctors, is it? He kept growing, and the pediatrician said “that child has a strange gaze,” but she didn’t know autism. He didn't know about high-functioning autism or Asperger's, so he didn't have any advice (ITA4)*

*I’m telling you, back in 1996 there wasn’t any kind of knowledge about it. Even now, but at least nowadays there is a bit of awareness. The child psychiatrist was a perfect example of a delinquent. No one explained anything to us, and nobody gave us any suggestions (ITA9)*

This causes a delegitimization of professionals, who end up being seen more as incompetent people in need of education than potential allies on a difficult journey.

However, Australian parents reported satisfaction with health professionals, who were recognized as experts in their field and seen as supportive and helpful. They complained about the lack of clear orientation by the health services. They missed someone giving them all the necessary information about the steps to follow once a diagnosis had been made, the services to turn to, the various therapies, the different types of professionals, etc. In this respect, they referred more to a structural deficit than to the individual limitations of individual professionals:

*I think there's some lack of communication with the process of what to do, like if I hadn't spoken to someone who already had an autistic child, I wouldn't have known what to do next (…). Like there is no sort of checklist, or anything like that, you know, so when you find out that your child is autistic, these are the things you need to go and do (…). If you are not proactive and you're not forward enough (…). I don't think they would give you that information by themselves (AU3)*

This lack of directions implies for parents the need to be proactive as well as the importance of knowing and talking with other parents of autistic children, who are recognized as a potential guide within a confusing and contorted field.

#### Autism and life: an overwhelming tunnel, a lifelong marathon

For both Italian and Australian parents, autism represented constant attention and continuous effort that made everything in their daily lives difficult or impossible. Autism seems to configure itself as an endless tunnel, a round-the-clock commitment, seven days a week, with no breaks, respite, or prospect of future resolution. Parents spoke of the fatigue that comes from the need to evaluate and choose everything carefully:

*The biggest difficulties are having to live constantly with this problem, not having the opportunity to distract oneself and do something else (..) the problem is that we don't have that phase where they are small now but then in a while we get free [she smiles], for us it'll always be like that, we'll always have a little one to look at (ITA1)*

*I'm trying you know to catch up with my friends (…) they are sort of getting to the next stage of their life when they are getting independent and I always constantly feel like the tired one who still has a toddler, you feel like you haven't really progressed in, in the sense of no matter what you do you feel like you'll never get it anywhere. And I think there's that constant exhaustion, which affects, constantly affects everything (AU5)*

Parents also express the frustration of feeling forever trapped in the same life stage without progress, changes, or evolution in the prospective future. In the above extract, the reiteration of terms referring to *constant* pressure shows the feeling of oppression and lack of a way out of the tunnel.

In the Australian sample, the experience of dealing with autism was also described as “a long journey,” “a marathon,” and a “work in progress” proceeding in small steps through progressive adjustments. Mistakes were described as part of a journey in which the important thing is to keep moving forward, saving energy for what has to come in this life-long condition.

### Parenting autistic children: reorganization of parents’ subjectivity and familiar dynamics

#### Parents’ positioning between self-sacrifice and self-empowerment

For the Italian sample, mothers felt positioned and tended to position themselves as real experts about autism and the ones charged with their child’s development. The figure of the sacrificial mother, who dedicates her life to the mission of caring for and treating her child, is crucial to reading women’s experiences in relation to the cultural context: “I went to read everything, I studied everything, that’s why then I did not work anymore, because by now I had dedicated myself to all these things” (ITA4). In relation to autism, the concept of sacrifice often implies an overlap between the maternal role and professional roles, such as therapist or teacher.

The greater amount of time mothers dedicated to the care and management of children also represented an issue for the Australian sample, as it affected mothers, especially in relation to their careers. As the following quote shows, women compare their conditions to their partners’, making evident the inequity between fathers and mothers and sometimes leading the latter to feelings of resentment.

*There's been a lot of resentment on my part in terms of career (..) you know we [my husband and I] were on the same salary, the trajectory was similar but he's gone from strength to strength and he's now in a multinational which is great for us because we all benefit from that (…) he gets the status and the recognition associated with that, whereas I feel that working part-time it's kind of hard (AU9)*

Both in Italy and Australia, parents also described the positive changes that having children with autism brought to their lives.

In particular, Italian parents highlighted how having to deal with autism has made them “better parents” as they had to develop particular skills to care for their children appropriately. This self-attribution differentiates parents of neurodivergent children not only from parents of neurotypical children but also from parents of children with other types of special needs. Nevertheless, it contributes to setting a sense of common destiny shared with the other parents of autistic children:

*We are all parents much … much better parents! It may sound presumptuous, but we have to because we have special children. Autism is a very special disability, it is not like a child who has a motor disability, he has a tough life too but it's different. Autism is a mental challenge for me and so being parents of children with these problems makes us, I don't want to say geniuses because we are not (…) mmh a step higher, I have often compared myself with other parents and we have an extra gear (ITA5)*

Australian parents described the multiple ways in which autism, besides making them better parents, has empowered their self-identity, helping them overcome personal limits. Parents reorganize their subjectivity to take the best out of a situation they are facing, even if they did not choose it. Most parents reported personal growth and changes with important implications for their identity.

*I used to be quite a shy person really, but I had to become quite a … pushy is the wrong word, but I've had to become quite a confident person who would speak out or he would not have had his needs met. That's a pretty big change for me personally and oh my goodness I'm so organized now because [laughs] I had to be (AU15)*

#### A lonely mother versus a family system

We saw significant differences between the two samples regarding the positioning of both parents and the configuration of the family system. Within the Italian context, mothers appeared to be lonely subjects; indeed, a strong sense of loneliness characterizes their experience from when the difficulties arose and all along the management of family life: “I’ve always been the one who had to take care of the child, always me seeing doctors, always me meeting with the teachers, always me going through this rigmarole” (ITA7). Mothers position themselves as “holding the weight of everything, the efforts, the decisions, the management of children,” feeling there were no real alternatives, as “it’s absolutely common for women to be the ones bearing the family’s struggles.” Fathers, thus, are positioned as those who “only see the tip of the iceberg of all this, they have no idea of all women’s everyday efforts (ITA1). Regarding the period prior to the release of the diagnosis, Italian mothers tended to position themselves as the only ones who were able to see a problem, in contrast with a skeptical social environment and a minimizing family context. Many of them reported having been labelled as “crazy,” “depressed,” “stressed,” or “overly apprehensive” by relatives and close ones, such as partners, who often identified them as the real problem and did not accept or understand the situation. In this context, it is easy for mothers to feel blamed and thus blame themselves.

*I started to tell my husband “Look at this child” and he said “no, it’s not him, it’s you, my sister has raised a child on her own, she has never complained about that, it’s you”. In short, it’s because you’re stressed, because you’re depressed. I was the depressed one (ITA4)*

Blaming was attached to almost every aspect of parenthood, which was perceived as constantly scrutinized and judged:

*In the end it is always the parents’ fault, no matter what they say, schools blame the parents, doctors blame the parents, the children blame the parents, so it’s always the parents’ fault, you do always wrong, so yes, you feel guilty (ITA11)*

Complementarily, Italian fathers were described by mothers as absent and often lacking interest in their child and their growth:

*I’ve always taken care of him by myself, my husband is very very busy since we are together and ehm maybe because the child was too lively, he gave it up, cause taking care of him was too hard. So he refused to be there, he didn’t take care of his son, he never took care of him (…) He never found himself in the extremely uncomfortable situations I’ve been (ITA7)*

Interviewed fathers also reported the feeling of being blamed, sometimes because of their traits ascribable to undiagnosed autistic disorder, sometimes for their educational methods:

*In the most difficult moments my wife told me that this [the child’s problems] happened because of me, that it was my fault. I might have had reactions or acted in ways she didn’t agree with, but I told her thousands of times it can’t be you’re always right and I’m always wrong! Anyway in the end our child’s expert is her, not me (ITA8)*

The Italian family system was then characterized by an absence of collaboration within the parental couple, whose bond is often weakened by a blame game activated by oppressive conditions.

Australian parents, however, tended to describe a more cooperative and united family environment, organized around the parental couple rather than the lonely mother.

Disagreements were also reported within Australian parental couples. However, parenting was overall described as shared, and autism as a condition affecting not only the single person but the couple’s relationship itself.

*My husband and I have just become a very efficient team (…) only twenty-five percent of marriages make it with an autism diagnosis But we were pretty determined from that very moment, we spoke about it a lot, that we were going to be the twenty-five percent and what that would take and from my perspective it took (AU9)*

Therefore, the emphasis was placed more on the relationship between parents, with their challenges, difficulties, and successes, rather than on the struggles of a lonely and solitary parent.

*Both my husband and I felt the same way, we were on exactly the same page. My husband and I would make sure that we have time together every week, we would go out or we would do something, we really make a conscious effort to have time together (AU5)*

Despite that, gender differences, stereotypical expectations associated with gender roles, and consequent disparities in parenting between mothers and fathers were still reported. Similar to what was reported by the Italian sample, Australian mothers tended to position themselves as much more committed to children’s daily care and reported having developed a more profound knowledge and understanding of their children compared to fathers.

*My husband and I are very lucky, we are great communicators with each other, we’re on the same page. However, it tends to be me who will go out and research and organize all the stuff our child needs for his extra needs. It’s me who’s gone, and I've educated myself about it all. Secondly, I’ve had to tell my husband everything I’ve learned and what we’re doing. I mean...but that’s a role I’ve taken on because I’ve decided not to work. We’ve decided as a family that I was going to drop back my hours and I was going to be the primary career as my husband couldn’t drop his hours. [...] That’s worked out really well but it’s also been hard (AU13)*

Differences also characterized the positioning of siblings. In the Italian sample, siblings are seen more as positive and valuable resources for the child with autism, as they represent a sort of “normative standard” with whom autistic children have to relate and deal with, thus offering interaction opportunities:

*Having three other children in a certain way is positive, because she has 3 siblings with whom she has to deal with (…) there are relationships that she has to carry on and problems that she has to face. On the other hand, it is true that … the others require energy too, so I can’t dedicate the whole day to her (ITA2)*

In the Australian interviews, instead, parents appeared to be more aware of and focused on the difficulties and repercussions that siblings have to face because of autism, such as lack of attention, the need to grow up quickly, psychological burdens, and life limitations.

*I have an older daughter who’s about to turn nine and I do at times feel sorry for her because I don’t think she gets the time she needs, and I particularly rely on her to help me, like if we go out I need her help (…) I feel a bit sorry for her, she had to grow up very quickly and be quite selfless in helping and things like that, I do try to give her enough attention, but I also see that she doesn’t get enough (AU4)*

### The unseen side of disability: autism communities between inclusion and segregation

#### Autism and social isolation

In both samples, parents, especially mothers, complained about the sense of isolation and loneliness they experienced as an effect of their children’s difficulties. The lack, or the loss, of social and friendship networks exacerbates the sense of being overwhelmed by autism.

Many parents attributed their social isolation to widespread ignorance, prejudices, and lack of understanding and acceptance of differences.

*I never had any problem with doctors, health services and teachers, I had problems with people (..) when I knew about autism, I told my friends and they just disappeared, they didn’t feel they could adjust, they felt uncomfortable to talk about their children with me, they all went away (…) they isolated me, they cut me out of their life, I found myself alone, totally alone! (ITA3)*

*You suddenly don’t have as many friends. You know, your children don’t get invited to places because they are different, and people will say that that’s not true, but it’s so true. It’s what happens. They don’t get invited to places, they don’t get the opportunity, they don’t get chosen for things because they socially stand out basically (AU6)*

The marginalization of children becomes the loneliness of the entire family, doubling the pain of parents.

Australian parents underlined that this situation allowed them to “bypass superficial friendship” (AU9) and surround themselves with understanding and welcoming people, valuing the relationships that have survived after the diagnosis or have been formed later.

In this regard, some parents, especially the Australian ones, found a new social network in what they call the “autism community,” that is, the community of families with neurodiverse children. Here, they found the “extra understanding” (AU13) and the social comfort they did not find in the social realm:

*I think what happens is that we end up with a circle of friends that are all part of the autism community, it becomes very difficult to have friends with children that are neurotypical because personally I feel, or I have felt over the years, sometimes it’s jealousy, sometimes it’s disappointment, but I felt that they couldn’t truly understand what we were going through and I’ve moved away from people that I felt didn’t understand (AU2)*

However, other parents problematized the exclusive or predominant belonging to “autism communities” as they see it as a clear risk of social segregation from “normal communities.” Attending activities and situations specifically designed for children on the autism spectrum may represent a safe and helpful space for families but also confront parents with feelings of exclusion from the broader society:

*I don’t want my daughter going to everything for autistic kids, ‘cause I think that is segregating her too. I think that her being included in the normal community too, is really really important. Not just having activities only for autistic kids all the time, and only going to those things with her (...) I still want my child to do things in normal community, because I think that’s how the community will accept children that have differences (AU3)*

*The idea is not to keep them there within a ghetto, but throw them into society giving them some crutches to get by. A nice thing is that he’s now attending this group that is mediated by a specialized educator, but the kids are not all disabled (ITA4)*

Thus, parents underlined the pressure to have their children included in open settings and contexts to train them to adapt to situations not designed for their specific needs and also as a way to prompt acceptance of differences.

#### Autism’s invisibility

For both Italian and Australian parents, the invisible character of autism was an aspect that makes its management in the social field even more difficult, as it increases the poor understanding of other people and, thus, the parents’ feeling of isolation. Indeed, the poor capacity of society to welcome people with autism is exacerbated by the fact that, in terms of outer appearance, autism is invisible, especially in the case of people with high-functioning autism.

Parents compared autism with other forms of disability that, being more physically visible and apparent, seem to receive more support and help. From their perspective, autism, being intangible and affecting more the behavior than the body, makes people turn away instead of helping:

*I just wish that there were a bit more understanding people...also with children who have disabilities also would feel the same but also because autism seems to be this invisible one, there’s no big sign or look or anything (AU4)*

*Their normal appearance is a problem. For me autism is a … a bastard disability, because you can’t see it, it’s not something you can see, like a physical impairment, so it can create higher barriers compared to other types of disabilities (ITA5)*

Italian parents highlighted that the invisibility of autism may be especially problematic in the school context. Indeed, the contrast between the children’s excellent performance in school and their social and relational difficulties is problematic in several ways. On the one hand, parents felt their kids’ high school abilities make their difficulties even more invisible and unseen. On the other hand, they saw schools as contexts that, being heavily focused on academic assessment and performance, cannot empower their children regarding social skills.

*You may not see the difficulties but you see the performance, that is, you see the 10/10 (very high grade) but you don’t see that he’s not able to interact with his classmates. The 10/10 is gratifying as you can say ‘ok school is fine’, but that 10/10 doesn’t make any difference, it doesn’t guarantee a good quality of life, cause if when he’s out of school he doesn’t hang out with anyone and locks himself at home … (ITA2)*

Australian parents connected the unseen difficulties of their children to the restricted range and poor variety of representations of autism in cultural products, such as movies or TV series.

*I think it would be great to better educate the community, so that they were aware a little bit more. I mean we educate people as we go. But just to be aware that not everybody with autism is Sheldon [character from the TV series The big bang theory] and not everybody with autism is Rain Man [movie]. There is a big spectrum in the middle (AU12)*

Stereotypical depictions do not fit the reality of parents’ lived experience and, according to the participants, do not contribute to spreading realistic information and educating the community about ASD.

## Discussion

Our research aimed to investigate the experiences of Australian and Italian parents of autistic children from an ecocultural perspective. We sought to understand how the conceptualization of problems may vary as socio-cultural conditions change. Our study employs a comparative approach, and the results reveal both commonalities and differences between the two groups of participants. Our discussion will begin with the latter, as it demonstrates that Italian and Australian parents, while encountering similar issues, express distinct interpretations of them that are connected to cultural differences.

In the first theme, which describes the pathway from symptom identification to diagnosis, it is observed that the Italian participants’ narrative is more indeterminate and uncertain, while that of the Australian parents is more precise and technical. The Australian parents evoke specific behaviors of their child, such as language use or relationship with food, to explain the origin of their suspicion of a possible disorder. According to our interpretation, the difference can be attributed to the Australian group’s higher expertise in symptom reading, which is linked to their higher educational qualifications. As a result, they apply a signifying frame that they have studied and deepened more than the Italians and use it to re-read their caregiver history. Therefore, one can observe a process of biographical reconstruction, which other research has shown in caregivers of patients with different disorders (Frigerio et al., 2013).

Once the diagnosis has been made, a further difference is observed in the relationship with health professionals. In the narrative of the Italian participants, there is often criticism of doctors who are accused of not being competent enough in autism. The criticism is directed at individual professionals, in contrast to what is observed in the Australian parents’ accounts. Indeed, the Australian parents’ accounts express criticism at a more general and systemic level, stating that practitioners provided them with much information but did not orient their choices concerning all the different information and treatment possibilities. This difference may be attributed to varying levels of trust in the health and care system. In Italy, there is low confidence in the social welfare system, causing individuals to rely on their relationship with a specific professional to seek answers to their problems and questions (Maturo, 2014; Camussi et al., 2023b). Critics may turn to that expert if this search is unsuccessful or unsatisfactory. In the Australian case, however, people have higher expectations of the system, which they trust more (Hardie and Critchley, 2008; Zhao et al., 2019). They expect the system to provide them with the necessary answers.

The second theme explores the impact of autism on family organization and parental subjectivity. These concerns relate primarily to the different positioning of mothers. The interviews with Italian mothers focused on the concept of the ‘sacrificial mother’, who invests a significant amount of time and resources to foster competence in autism. This is perceived as necessary to compensate for the competence deficits in health professionals and ensure the best possible treatment for their children. In the case of Australian mothers, the participants’ discourse is focused on the figure of the ‘penalized mother,’ who is forced to give up more satisfying jobs or career prospects to care for her children. In both contexts, maternal positioning appears to be linked to gender stereotypes that assign mothers the most significant responsibility of childcare. However, in the Italian group, this stereotype seems to be shared by the mothers and legitimized in a familistic culture that takes women’s moral obligation to care for granted (Ruspini, 2015). As a result, these mothers present their commitment and sacrifice as valuable elements to be claimed. Regarding Australian mothers, they denounce unfair treatment due to the unequal distribution of burdens, which they believe is not culturally legitimized despite being their reality. A paradox arises in this situation: the Italian participants report less frustration because they find meaning in their sacrifice by accepting the normative reference they have internalized.

When focusing on the family, another difference emerges in the role attributed to siblings. The Italian participants predominantly discuss the advantages of sibling relationships for children with autism. In their perspective, siblings served as a normative standard for social adjustment and provided a valuable learning opportunity. For the Australian participants, the focus is on the risks that siblings run since, to best focus on the needs of the child with autism, parents may end up neglecting them. In both cases, therefore, the discourse seems to revolve around the issue of siblings’ normality, which appears to be a resource in the case of the Italian participants, whereas it is at risk in that of the Australians. This difference can be attributed to a different representation of the context and family relationships. In the Italian one, it is widely believed that parents make sacrifices for the well-being of their children and that children, in turn, are willing to invest time and resources in the family system when needed, for example, to help cope with the various effects of a sibling’s autism (Reher, 2004). The family bond is based on mutual duties that hold people together. In Australian culture, children are expected to develop independence from their parents and become autonomous (Haslam et al., 2020). Therefore, there is a concern that the desire to collaborate in the care of a child with autism or the diminished responsiveness of the parents may hinder this pathway.

A difference between the two groups is evident in their approach to social relationships. Australian participants tend to seek out and participate in a community of parents who share the same experience, socializing their problem as parents of a child with autism. This search for new opportunities for socializing leads to discovering new positive resources. In the Italian group, however, individual paths are described more frequently, indicating a greater distrust in the community dimension and resulting in less involvement in parental group situations. Italian parents expect to be included more in their usual networks of relationships, such as family and lifelong friends, and express dissatisfaction when this does not happen sufficiently. In Italy, limited geographical mobility results in stable relationship networks that can last individuals’ entire lives (Tomassini et al., 2003). This is because school friends, cousins, siblings, and even the children of parents’ friends often form the most substantial ties (Ruspini, 2015). The relationship is a vital identity element, making it difficult to detach oneself and open up to new experiences, even when traditional networks are not supportive. In contrast, Australia has greater geographical and labor mobility (Watson, 2020), allowing people to build new friendship networks more easily. Relational mobility implies that choices can be made based on commonalities of interests, experiences, and problems, which may be temporary but satisfying or quickly abandoned when not (Thomson et al., 2018). This allows for greater flexibility.

Despite the differences between the two groups, our thematic analysis reveals that there are also significant similarities in the parental experience. The first theme highlights positive aspects of autism, such as parents’ references to their children’s precocity, sensitivity, and unique abilities that allow them to perform specific tasks better than their peers. This representation can be interpreted as a compensatory measure or a defensive tactic (Ashforth et al., 2007) against the social stigma that parents may feel. However, it allows parents to highlight a positive aspect that provides a more comprehensive and nuanced image of their children. A common element that is also relevant is the portrayal of the diagnosis as a liberating moment, allowing individuals to articulate various symptoms and behaviors that had previously been difficult to understand in comprehensible, communicable, and manageable terms. Significant similarities also exist in terms of caregiving for both groups. It can be an exhausting experience characterized by constant uncertainty about the best therapeutic and educational practices to adopt. This creates an emotional and physical burden for parents. However, the experience of this impactful journey is not presented solely in negative terms. Both groups of participants noted a positive change in their parental identity. The parents interviewed reported feeling like better parents due to the skills they developed in caring for their child diagnosed with autism. Finally, both groups share the perception of an unwelcoming, if not explicitly stigmatizing, context that contributes to the development of loneliness among these parents.

These elements, which are common to both groups of participants, have already been identified in the literature on the parental experience of autism, as presented in the introduction to this article. Despite much research devoted to identifying and analyzing these themes and problems in different contexts over many years, they remain unresolved. Although various studies have usefully illuminated the difficulties faced by parents of children with disabilities and shown their social implications, they have not yet succeeded in fostering a relevant change in the provision of services, the definition of policies on the subject, or the widespread culture surrounding this phenomenon. This issue does not stem from the limitations of a single study or the peculiarities of a national context but rather from the actual impact these studies achieve, even if they have identified individual and collective discomfort. The effect of these studies, including the one presented here, appears to be limited to the researchers conducting them and the participants who are given a voice. During their interactions with us, individuals can positively reframe their experiences. This is supported by the feedback we receive at the end of interviews or when discussing research results. However, it remains unclear how research can effectively impact social and political change in health culture and practice. Because we value research with these parents and support their challenges, we believe it is essential to address this question explicitly in the final discussion. This will open up new paths for future work together. From a clinical perspective, this study highlights the importance of supporting the entire family system in managing the challenges associated with autism rather than just focusing on a single parent, typically the mother. Health professionals should be aware of the gender stereotypes that still exist and create disparities between mothers and fathers. It is crucial to consider the negative impact of implicitly expecting mothers to take on the role of co-therapist. Additionally, this research points to the need to support more positive relationships between families and health services, providing parents with the necessary information and guiding and orienting them along the network of opportunities and resources available to them to face their difficulties. Another focus of intervention is the so-called ‘autism communities’. According to parents, these communities can be both a resource for social inclusion and a sign of social segregation from the rest of the community. It is essential to design situations and activities that are inclusive for autistic children without being perceived as marginalizing by families. In this regard, health professionals and social workers must acknowledge and address the concerns of parents who feel that the label of autism is overshadowing their entire lives and identities.

Future research should extensively address the similarities and differences between cultural contexts. To better understand how autism is perceived and experienced in socio-cultural contexts, research directions in this area should include more cross-cultural and comparative studies from Western cultures and non-Western countries. This will help to shed light on the impact of expectations and demands on childhood, parental roles, and gender inequalities.

### Limits

This study is not without limitations.

Due to the constraints of available resources, the number of participants involved in this research in each country is limited. Nevertheless, the potential for comparing these two distinct contexts is a compelling aspect of this study, particularly given the dearth of analogous studies. The Italian parental associations were national in scope and based in the country’s main northern city. Consequently, the sample was drawn from northern Italy, while the Australian sample spanned a broader area in the country’s southeast. Despite the difference in the extent of the two geographical areas, taking into account the differing population density, which is lower in Australia than in Italy, there is no relevant disparity in terms of catchment areas.

Secondly, the interviews were conducted in person in Italy, as the participants were more readily accessible to the interviewer. Conversely, all interviews in Australia were conducted via telephone due to the greater geographical distances involved.

A further limitation of the research is that the two samples are not numerically homogeneous. This is due to the differences in recruiting participants, as the Italians were asked for availability in person for an interview lasting around an hour, which made it more difficult and time-consuming to involve them in the research. Additionally, some Italian parents declined our invitation to participate, citing a lack of immediate benefit from the study.

Notwithstanding these limitations, the use of a common interview outline, the involvement of a single, experienced interviewer in all interviews, the experiential orientation of the interview moderation, and the detailed narratives collected collectively render the two datasets comparable.

## Data availability statement

The raw data supporting the conclusions of this article will be made available by the authors, without undue reservation.

## Ethics statement

The studies involving humans were approved by University of Milano-Bicocca Ethics Committee and Deakin University Human Research Ethics Committee. The studies were conducted in accordance with the local legislation and institutional requirements. The participants provided their written informed consent to participate in this study. Written informed consent was obtained from the individual(s) for the publication of any potentially identifiable images or data included in this article.

## Author contributions

AF: Conceptualization, Data curation, Formal analysis, Funding acquisition, Investigation, Methodology, Project administration, Writing – original draft, Writing – review & editing. TK: Conceptualization, Formal analysis, Methodology, Supervision, Writing – original draft, Writing – review & editing. RR: Writing – original draft, Formal analysis. ES: Formal analysis, Writing – original draft. EC: Writing – original draft, Writing – review & editing, Funding acquisition. LM: Conceptualization, Formal analysis, Funding acquisition, Methodology, Supervision, Writing – original draft, Writing – review & editing.
