# Supplementary material for: Experiences of parenting for autistic children in Australia and Italy: a qualitative cross-cultural comparison
Source: Front Psychol. 2024 Aug 5;15:1409234. doi: 10.3389/fpsyg.2024.1409234 (PMC11330893; doi:10.3389/fpsyg.2024.1409234)
Supplement: Supplementary file 1 [file Data_Sheet_1.DOCX]

# Interview Schedule

**UNDERSTANDING THE PARENTAL EXPERIENCES OF AUTISM**

***Introduction***

The researcher will begin with an introduction:

My name is … and I am conducting this interview as part of a research project to further understand the experiences, perceptions and opinions of parents with a child diagnosed with Autistic Spectrum Disorder. I would like to thank you for participating in our research.

Before we get started, I would just like to remind you that participation in any research study is voluntary and you are free to withdraw at any time, without explanation. In addition, you do not have to answer any questions that you do not wish to. Our discussion today is confidential and the interview data will be stored on a secured computer server only accessible by the research team.

To start with, I’d like to ask you some medical and demographic questions.

***Medical and Demographic Questions***

1. What is your age?
2. What is your country of birth?
3. What is your child’s gender?
4. What is your child’s current age?
5. When was your child diagnosed with autistic spectrum disorder?
6. What is your child’s position on the autistic spectrum?
7. What is your marital status?
8. What is the highest level of education you have completed?
9. What is your current employment status?
10. What was your employment status prior to diagnosis?

*Following collection of medical and demographic data, the interview will be conducted in a semi-structured manner with the following questions used as a guide.*

***Questions to guide semi-structured interview***

(note: for each aspect, do prompt the telling of examples, anecdotes, and significant events)

1. Can you tell your story as a parent of a child with Autistic Spectrum Disorder, from when you first started to notice signals, up to when your child was actually diagnosed?

Prompts:

- Can you describe your emotions at this time?
- What was helpful during this time?
- What was unhelpful at this time?

1. Did you have any personal ideas about the possible reasons of your child’s difficulties?
2. Can you describe your initial reaction to the diagnosis?
3. How has your child’ diagnosis been affecting your life?
4. How has your child’ diagnosis been affecting your interactions with your partner and family?
5. How has your child’ diagnosis been affecting your interactions with friends and the broader social context?
6. What have been the greatest problems that you had to face in relation to the difficulties of your child? How have you dealt with them?
7. Can you tell me a little bit about how you see yourself since your child’s diagnosis? Has your view of yourself changed since your child received the diagnosis?
   1. [If the response is that the participant’s sense of self has changed the researcher might ask] Can you reflect on some of the reasons for the change in your sense of self?
8. I’d like to ask you about the type of care and support your child, you and your family have received.
   1. What type of treatment did/does your child and you as a parent receive?
   2. How have you oriented yourself in choosing the best treatment for your child?
   3. Did/does the support that your child, you and your family received meet your needs? Why?
9. As parent of a child with autism, which types of health care professionals have you interacted with? Prompt: general practitioners, paediatricians, child psychiatrists, psychiatrists, psychologists, social workers
10. What type of relationship have you had with health care professionals and services?
    1. What aspects of your child and your family care do you feel have been adequately addressed?
    2. What aspects of your child and your family care do you feel have not been adequately addressed?
    3. What could the health care professionals and services do to better support parents of children with autism?
11. As parent of a child with autism, what type of relationship have you had with schools and teachers?
    1. What aspects of your child care do you feel have been adequately addressed by the school system?
    2. What aspects of your child care do you feel have not been adequately addressed by the school system?
    3. What could teachers and schools do to better support parents of children with autism?
12. How do you imagine your child as an adult when you will might not be able to help him/her everyday?
13. What could the broader community do to better support parents of children with autism?
14. Is there anything else that you would like to share about your experience as a parent of a child with autism?

***Conclusion***

Thank you for sharing your experiences; it is very much appreciated.

After the interview has been transcribed, would you like to receive a copy of the transcript to read through to see if it has captured all aspects of your experience that you feel are important to share?

*If yes:* We will email you a de-identified copy of the transcript that will be secured by a password. We will telephone you to provide you with the password.
